# Simulation of Depth of Wear of Eco-Friendly Concrete Using Machine Learning Based Computational Approaches

**DOI:** 10.3390/ma15010058

**Published:** 2021-12-22

**Authors:** Mohsin Ali Khan, Furqan Farooq, Mohammad Faisal Javed, Adeel Zafar, Krzysztof Adam Ostrowski, Fahid Aslam, Seweryn Malazdrewicz, Mariusz Maślak

**Affiliations:** 1 Department of Structural Engineering, Military College of Engineering (MCE), National University of Science and Technology (NUST), Sector H-12, Islamabad 44000, Pakistan; moak.pg18mce@student.nust.edu.pk (M.A.K.); adeel.zafar@mce.nust.edu.pk (A.Z.); 2 Department of Civil Engineering, CECOS University of IT and Emerging Sciences, Peshawar 25000, Pakistan; 3 Military Engineer Service (MES), Ministry of Defence (MoD), Rawalpindi 43600, Pakistan; 4 Faculty of Civil Engineering, Cracow University of Technology, 24 Warszawska Str., 31-155 Cracow, Poland; krzysztof.ostrowski.1@pk.edu.pl (K.A.O.); mmaslak@pk.edu.pl (M.M.); 5 School of Civil and Environmental Engineering, National University of Sciences and Technology (NUST), Sector H-12, Islamabad 46000, Pakistan; 6 Department of Civil Engineering, Abbottabad Campus, COMSATS University Islamabad, Abbottabad 22060, Pakistan; arbabfaisal@cuiatd.edu.pk; 7 Department of Civil Engineering, College of Engineering in Al-Kharj, Prince Sattam Bin Abdulaziz University, Al-Kharj 11942, Saudi Arabia; f.aslam@psau.edu.sa; 8 Department of Materials Engineering and Construction Processes, Wroclaw University of Science and Technology, Wybrzeże Wyspiańskiego 27, 50-370 Wroclaw, Poland; seweryn.malazdrewicz@pwr.edu.pl

**Keywords:** fly-ash, depth of wear (DW), abrasion resistance, artificial intelligence (AI), random forest regression (RFR), gene expression programming (GEP)

## Abstract

To avoid time-consuming, costly, and laborious experimental tests that require skilled personnel, an effort has been made to formulate the depth of wear of fly-ash concrete using a comparative study of machine learning techniques, namely random forest regression (RFR) and gene expression programming (GEP). A widespread database comprising 216 experimental records was constructed from available research. The database includes depth of wear as a response parameter and nine different explanatory variables, i.e., cement content, fly ash, water content, fine and coarse aggregate, plasticizer, air-entraining agent, age of concrete, and time of testing. The performance of the models was judged via statistical metrics. The GEP model gives better performance with R^2^ and *ρ* equals 0.9667 and 0.0501 respectively and meet with the external validation criterion suggested in the previous literature. The k-fold cross-validation also verifies the accurateness of the model by evaluating R^2^, RSE, MAE, and RMSE. The sensitivity analysis of GEP equation indicated that the time of testing is the influential parameter. The results of this research can help the designers, practitioners, and researchers to quickly estimate the depth of wear of fly-ash concrete thus shortening its ecological susceptibilities that push to sustainable and faster construction from the viewpoint of environmentally friendly waste management.

## 1. Introduction

Concrete is the most widely used construction building material in the world [1]. It is expected that the annual consumption of concrete lies around 23 trillion kilograms [2]. Ordinary Portland cement (OPC) alone produces extensively carbon dioxide (CO_2_) and has a malignant effect on the environment and energy consumption on resources [3]. Moreover, the cement industry is one of the main producers of greenhouse gases (GHG) in the atmosphere. Researchers have adopted potential ways to mitigate the adverse effect of traditional concrete on the environment and to make environmentally friendly concrete by using waste materials [4,5,6,7,8]. These waste materials not only enhance the fresh and hardened properties but also give durability as well as enhanced effect to the concrete. Moreover, their utilization as supplementary in cementations composite gets rid of the disposal issue in the land [9]. Fly ash (F) is a secondary waste by-product that is generated by using powered coal as petroleum in thermal plants. Part of the ash during the burning in the production stage is flown out from the flue chamber to the atmosphere with the emission of heat and remaining settled at the bottom of the kiln base [10,11,12]. These residual ashes have cementations characteristic due to the presence of silica and aluminum content. Their use on the concrete side reduces shrinkage, improves fracture toughness and frost resistance, and ultimately enhances the narrow interfacial transition zone between heterogeneous phases.

**Figure 1:**
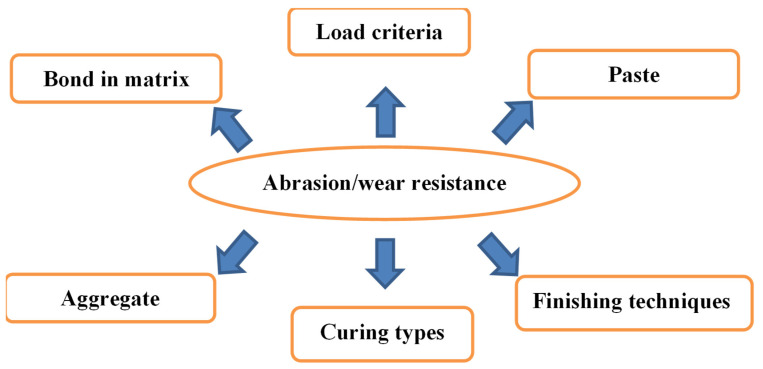
Parameters affecting abrasion resistance of concrete.

The concrete strength of the reinforced structure is evaluated by compressive strength (CS) [13]. However, the durability aspect is also a prerequisite criterion as concrete structures are vulnerable to damage due to wear and severe environmental conditions [14]. The abrasion resistance is also one of the main aspects and is often perhaps more vital than the concrete compressive strength when the load acts directly on the surface like in the construction of floors, industrial enterprises, airport runways, spillways of dam, hydraulic structures, parking lots, concrete roads, tunnels, among others [15,16,17,18]. It occurs due to wearing, mechanical scraping on the surface of the concrete. Moreover, concrete and mortar resistance in terms of wear is dependent upon material, load, strength, and hardness of surface as presented in Figure 1. The concrete abrasion resistance is directly related to curing time, compressive strength, and material properties irrespective of cement replacement by filler materials [19,20]. To achieve satisfying abrasion resistance, emphasis and attention are given on sample preparation. Strong specimen yield better abrasion resistance which ultimately enhances the durability of concrete [21].

Zhen et al. [22] studied the abrasion resistance and microstructure of concrete by cement replacement with filler materials. The author reported maximum enhancement in strength with the refinement of pore size which ultimately increases the abrasion resistance of concrete. Jain et al. [23] studied the microstructure and abrasion resistance of concrete with shredded plastic waste bags by fine aggregate replacement with percentages. The author reveals a significant decrease in density, compressive strength, flexural strength, and workability. However, enhancement in abrasion resistance, energy absorption, and impact resistance is observed in plastic waste concrete specimens. Adekunle et al. [24] investigated the effect of abrasion resistance and compressive strength by varying exposure contact, concrete age, and materials constituents. The author observed that compressive strength is directly related to abrasion resistance. Moreover, fine aggregate by 28.7% by binder mass show worst performance, whereas coarse aggregate by 45% cement mass gives the best performance for compressive strength and abrasion resistance of concrete. Wang et al. [25] examined the effect of varying dosages of silica fume, PVA fiber and their combination on compressive strength, tensile strength, abrasion resistance, volume stability, and drying shrinkage of concrete. Significant enhancement is observed in the mechanical properties of concrete with abrasion resistance by the addition of silica fume and PVA fiber. However, the addition of PVA fiber reduces the drying shrinkage and increases the anti-cracking resistance of concrete and cement pastes. Jain et al. [26] investigated the fresh and hardened properties of self-compacting concrete SCC by varying granite waste (GW) as a fine aggregate replacement with different percentages. The author observed that varying dosages of GW as replacement of fine aggregate has a profound effect on abrasion resistance and compressive strength of SCC. However, GW with 40% replacement gives significant enhancement results in overall properties. Yildizel et al. [27] examined the mechanical properties and abrasion resistance of roller-compacted concrete by the addition of waste glass fiber (WGF). A different percentage of WGF is added by volume to evaluate the strength mechanism. However, adding 2% by volume in reinforced concrete mix yields maximum. Nazari et al. [28] investigated the effect of nano-size particles on concrete by changing curing temperature. The author concluded that the inclusion of small size particles in the cement matrix increases the abrasion resistance and compressive strength of concrete. Similarly, Hui et al. [29] investigated the effect of polypropylene fibers (PP), nano-silica (SiO_2_), and nano titanium oxide (TiO_2_) on the abrasion resistance of pavement concrete. Significant enhancement is observed in the matrix by the addition of nano media as compared to PP fibers. Moreover, TiO_2_ gives a better response against overall abrasion in the matrix. Chernysheva et al. [30] investigated the effect of scientific pattern of Thermal-Power-Plant (TPP) waste on the microstructure of gypsum cement binder. Their findings reveal that combining gypsum-cement binder and TPP waste increases the physical and mechanical characteristics. Tang et al. [31] examined the fracture behavior of rubber modified recycled aggregated concrete (RRAC) at varying temperatures (200, 400 and 600) °C. The finding of their study shows that the rubber aggregates have greater unstable fracture toughness than the initial cracking toughness of recycled aggregate concrete after exposing to elevated temperature. Yunchao et al. [32] studied the mutual effect silica-fume and nano-silica on the cubic compressive strength, split tensile strength and stress strain relationship of recycled aggregate concrete. A good pozzolanic activity of silica fume and nano-silica was reported and 6% silica-fume and 3% nano-silica were recommended as supplementary cementitious material for recycled aggregate concrete. Abdul Aziz et al. [16] examined the abrasion and skid resistance of pavement concrete by utilizing waste PP and palm oil fuel ash (POFA). Specimen with various dosages of PP and POFA as cement replacement was prepared. The author reveals that the intrusion of PP fibers in the concrete matrix decreases the compressive strength of the overall matrix by 17%. However, enhancement in abrasion and skid resistance by 25% is observed as compared to the control specimen. Moreover, POFA improves the matrix properties due to the pozzolanic nature in samples. Likewise, the authors [33,34] observed that copper slag as fine aggregate replacement in concrete improves the slake and abrasion resistance. Malazdrewicz et al. [20] used ANN algorithm for the prediction of depth of wear of cementitious matrix with fly ash. The result reveals that ANN give a good linear model with R^2^ = 0.997 for testing, R^2^ = 0.998 for learning and 0.995 for validation. Similarly, Malazdrewicz et al. [35] employed same technique and achieved a good correlation with experimental and prediction results.

Concrete is made from heterogeneous materials that include the paste phase and aggregate phase [36]. Table 1 shows the effect of waste material on properties of concrete. The abrasion resistance of concrete is time-consuming and is purely based on the surface test. The heterogeneity of the matrix such as sedimentation of coarse aggregate, porosity of the upper layer of concrete (mainly composed of cement paste and fine aggregate) and the ITZ transition zone, chemically and microstructurally different then the bulk binder phase altogether creates uncertainty of measurements. Moreover, these methods require a field sample taken from the site and then used to check the abrasion resistance in the laboratory. Furthermore, a sample taken from the field sometimes deteriorates during placement due to unavailability of equipment in the field. For this reason, separate samples are sometimes created specifically for laboratory purposes. However, they may differ from the element that needs to be examined in terms of properties. It is also worth noting that the current methods of testing abrasion resistance do not reflect the real conditions of abrasive wear in some structures. Their disadvantages include the relatively long duration of research, and the long time to obtain the results. Generally, physical measures are carried out to obtain compressive strength and abrasion resistance. This is achieved by testing various cubes and cylinders with mixed design ratios in the laboratory. Thus, efforts have been made to predict the strength, elastic modulus, and many properties of the concrete specimen by using regression and machine learning models. This will not only reduce the uncertainty in the prediction aspect but will give the required quantities in mix design to give strength. These supervised machine learning algorithms give an adamant relation and predict the best model using a set of input variables as illustrated in Table 2.

## 2. Significance of the Study

The main objective of the research is to utilize the GEP (part of machine learning (ML) technique) with random forest regression (RFR) an ensemble modeling approach via Anaconda Python to estimate the abrasion resistance (depth of wear) of fly-ash-based concrete. An RFR that involves a bagging mechanism is used for optimization through developing 20 sub-models to deliver a higher coefficient of determination (R^2^). To assess the suitability of models, the K-fold cross-validation (KFCV) and statistical error metrics such as root mean square error (RMSE), relative root mean square error (RRMSE), mean absolute error (MAE), relative squared error (RSE), and performance index (*ρ*) are used. The models are also tested via the statistical metrics suggested in the literature. In the end, the Anaconda Python-based permutation feature analysis is conducted.

## 3. Description of Collected Experimental Data

The data points used for modelling the abrasion resistance of concrete have been taken from published research work [20] (See Appendix A1). The performance and reliability of the model depend on the total number of instances used for modelling. The database includes total 216 experimental test results with depth of wear (DW_mm) as response parameter and nine different explanatory variables, i.e., cement content C (kg/m^3^), fly-ash F (kg/m^3^), water content W (kg/m^3^), fine aggregate FA (kg/m^3^) coarse aggregate CA (kg/m^3^), plasticizer P (kg/m^3^), air-entraining agent (AE_kg·mL/m^3^), age of concrete (A_days), and time of testing (T_min). The interdependency of the selected variables needs to be checked to avoid complications in the interpretation of the model. This problem of correlation between selected variables is referred to as multicollinearity. To counter this problem, the coefficient of correlation between two explanatory variables must be less than 0.8 [66]. It can be seen in Table 3 that the correlation coefficient (negative and positive) of all selected variables is significantly less than 0.8. This dictates that no multicollinearity problem between the selected variables and can be effectively used for the development of a generalized model. All the chosen variables greatly influence the abrasion resistance of concrete.

### Python Based Programming for Presenting Data

**Figure 2:**
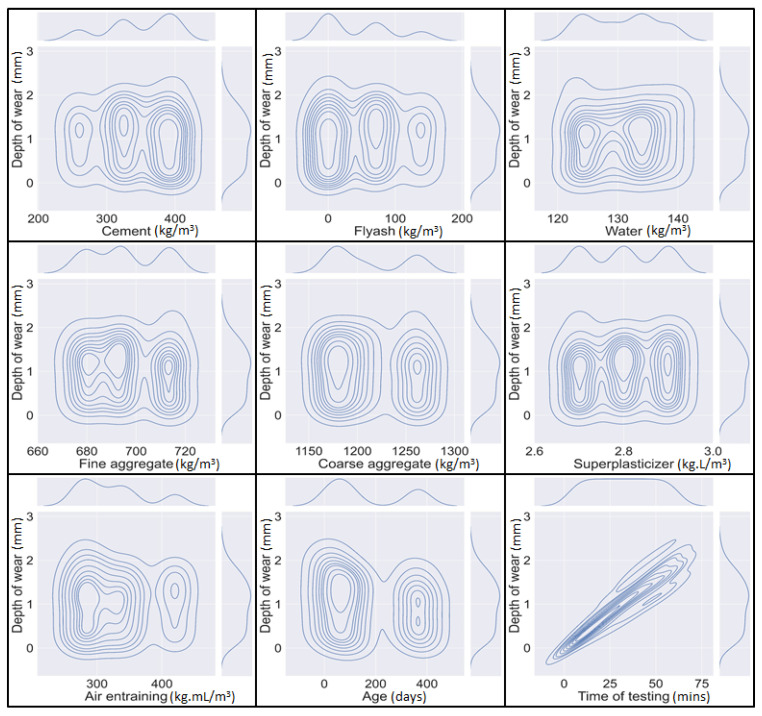
Python-based contour plot between explanatory and response parameter.

For a more significant illustration of the data an Anaconda-based python programming, version 3.70 was utilized. The effective performance of the model depends on the distribution of the explanatory variables [67]. A Python measure was used to check the distribution of selected variables and to choose the optimum dosage and their influential impact on the abrasion resistance of concrete. Figure 2 illustrates the contour plot that shows the relation between the chosen variables and their distribution. The relative maximum spread of the data set to abrasion resistance is represented by the rectangular segment of the graph. The maximum contour lines depict the high density of every selected variable to the abrasion resistance of concrete. Their frequencies are appropriately higher. It must be noted, that a good model can be obtained if explanatory variables have high frequencies [68,69]. Thus, these variables can be taken for the accurate and effective prediction of the abrasion resistance. For a more meaningful interpretation of the selected data set, the statistic of response and explanatory variables is presented in Table 4. That makes the explanation of the data very easy. It includes the distribution shape (kurtosis and skewness), center of data points (median, mean, and mode), extremes of data points (minimum and maximum), and spread of the data (standard deviation). The data for a particular variable is right-tailed (positive skewness) if a high concentration of data is to the left of the mean and left tailed (negative skewness) if a high concentration of data is to the right of the mean. Moreover, it is exactly zero for asymmetrical shape. The kurtosis dictates the flatness or peakedness of the data in relation to normal distribution. The leptokurtic or positive kurtosis dictates that the data is more peaked than the normal distribution and platykurtic or negative kurtosis shows that the data is flatter than the normal distribution. Generally, the acceptable range of skewness and kurtosis is −3 to +3 and −10 to +10 respectively [70]. The development of an effective, reliable, and accurate model is greatly dependent on the variables [71].

## 4. Research Methodology

Various researchers utilized machine learning methods in different fields to forecast and comprehend the properties and behavior of the materials. In this study, two different machine learning techniques namely; random forest regression (RFR) technique and gene expression programming (GEP) are used to estimate the abrasion resistance of concrete. These methods are chosen because of their effectiveness and robustness in predicting outcomes in related works, as well as their position as the leading data mining techniques.

### 4.1. Random Forest Regression Approach

Shapire et al. [72] and Breiman et al. [73] introduced two different ensemble learning methodologies known as boosting and bagging of classification trees respectively. In boosting methodology, the consecutive trees are used to give additional weightage to the nodes that has been predicted incorrectly by previous trees. In last, the weighted vote is selected for estimation of output. While in bagging methodology, the consecutive trees are independent on the previous predictor. Every tree is constructed independently via bootstrap samples of data, and finally, the majority vote is chosen for estimation of outcome.

Brieman [74] proposed a modified bagging technique known as random forest regression (RFR). It can be used as both a classification and regression technique. In RFR, an additional layer of randomness is added for the bagging mechanism. While creating trees via a bootstrap sample of data, RFR alters the way of regression trees as compared to standard trees. In RFR, every node is divided via the finest predictor from a subclass of predictors picked at random at that node. RFR seems like a counterintuitive approach that gives an outburst performance as compared to support vector machine (SVM) and neural networks (NN) and is vulnerable to overfitting [74]. Furthermore, RFR quite easy to use in that it only has two hyper-parameters (the variables in the random sample set at nodes and the trees in the forest) and is generally insensitive to their values. In the recent decade, a lot of researchers used RFR for the prediction of different properties of concrete [69,75]. The following steps are involved in the RFR algorithm;

1. For each tree, two-third of the whole data is selected at random, known as bagging. Variables for prediction are selected at random, and the best split on such variables is used for dividing the nodes.
2. The out-of-bag (OOB) error is calculated for all trees via the one-third data. The OBB error is aggregated from every tree to measure the ultimate rate of OBB error.
3. Each tree in the forest generates a regression and the model chooses the trees with the most votes from the forest. Votes can be either 1’s/0’s. A prediction probability is identified as the percentage of 1’s received.

### 4.2. Gene Expression Programming

**Figure 3:**
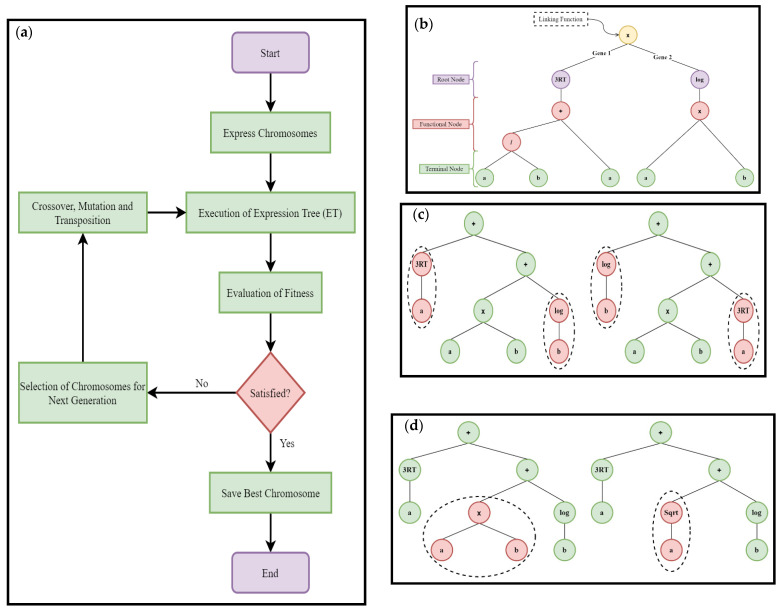
(**a**) Main steps involved in GEP algorithm; (**b**) Representation of typical expression tree (ET); (**c**) Process of crossover; (**d**) Process of mutation taking place in GEP.

The gene expression programming (GEP) is an extended version of genetic programming (GP) and genetic algorithm (GA), which works on the selection of population chromosomes and evaluates them according to the selected fitness condition, and performs the genetic alteration using one or more genetic operators. It is the type of evolutionary algorithm which executes both the fixed-length linear chromosomes replicated from GA and representation of expression parse trees like the structure of different shapes and sizes from GP [75]. The Karva language is used as a programming medium which is similar to list processing (LISP) language. The flow diagram illustrated in Figure 3a covers the main phases involved in the execution of the GEP algorithm. The presented process is recurred many times until the refined solution is acquired [76]. The ETs in GEP illustrate different complications which include constants, functions, operators, and variables. A single ET is composed of a root node, functional node, and terminal node. The mathematical expression for the ET presented in Figure 3b has two genes with multiplication as a linking function and can be written as Equations (1)–(3).

(1) $$Gene\;1=\sqrt[{3}]{\frac{a}{b}+a}$$

(2) Gene 2=log(a×b)

(3) Prediction=Gene 1×Gene 2

To expand and evaluate the fitness efficiently, the random portion of the parent tree is substituted with another arbitrarily selected portion, known as crossover as illustrated in Figure 3c. The crossover has two off-springs, both include the genetics of their parents. Figure 3d shows the mutation takes place at a node where an arbitrary component of the parent tree is substituted by a newly created component or part of the parent tree [77].

The research revealed several advantages of GEP in comparison with other classical regression techniques. Classical regression techniques use some pre-defined functions while GEP reflects the initial non-linearity without considering the pre-defined functions. Therefore, GEP is considered to be more efficient than other regression approaches and neural networks for modeling and obtaining mathematical relations for experimental research of multivariate problems [78]. There is no specific procedure for achieving the best result with a given set of parameters to optimize the accuracy. This adds to the complexity and execution time of the model development. To avoid this problem, this study used the most effective parameter values in GEP to obtain the most reliable and less complicated relationship. The selection of such effective hyper-parameters depends on the suggestion in previous research and the hit and trial process. To increase accuracy and to achieve the best model, initially, the lowest parameter level referring to one gene was utilized, afterward, different parameters were assessed to train the model. In GEP different linking functions are available like subtraction, multiplication, addition, and division. To resolve a problem, choose one gene chromosome, and then continue with the modeling by increasing the head size. Even so, the number of genes can be increased considerably and a function can be chosen to connect the branches of the expression trees [78].

The complex nature of the model cannot be assessed. In addition, the difficulty of a model increases as the number of inputs increases. Regarding this, the higher the genes, the higher will be the model complexity. Increases of other parameters value have a similar effect on the outcome. One cannot exactly define the complexity. Another source of complexity is the creation of a new generation to achieve a higher-level regression and more suitable fitness when training the data sets. The hyper-parameter tuning of GEP algorithmic space is based on the recommendations in previous research and the hit and trial process [78]. The GEP algorithmic space involves the following steps;

1. Choosing a fitness function allowing the GEP to achieve an optimum solution by itself. Here, a fitness function equals to 1000 was used [79].
2. Choosing a set of terminals that involve the explanatory variables considered for the prediction of response. This study uses nine different explanatory variables for the prediction of wear depth of concrete (explained in Section 3).
3. Choosing the set of functions. To get a simple GEP equation, this research uses four basic arithmetical functions, i.e., +,−,×, and /.
4. Choosing the architecture of chromosomes, i.e., the genes, head size, and linking function. To avoid complexity, in this research three genes with head size equal to ten and addition as a linking function were utilized.
5. Choosing the set of genetic operators. A mixture of crossover, mutation, and transposition was used as a genetic operator.

### 4.3. K-Fold Cross-Validation (KFCV) and Statistical Metrics

**Figure 4:**
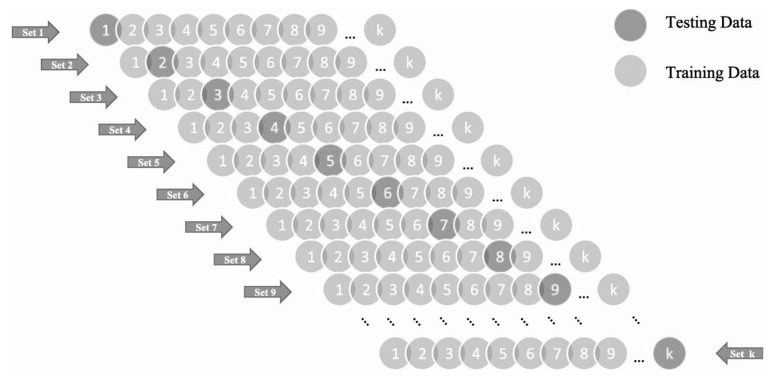
Flow diagram of K-Fold cross-validation algorithm.

K-fold cross-validation (KFCV) algorithm is as part of Jack’s knife test that is usually used to reduce the biases in a random sampling of the training set and hold out data set and to avoid the overfitting complexity. It splits the whole data set into k-subsets of data. 1-subset is hold-out for testing and the rest k-1 subsets are used to train the model [80]. Kohavi’s [81] research indicated that the stratified ten-fold validation produces accurate variance and is best for the optimum computational time. This study uses the same ten-fold to assess and validate the performance of the model. The entire data is split up into ten different subsets. In all ten generations of model creation and validation, it selects a different subset of data for training while testing of the model is executed with other subsets of data. As can be seen in Figure 4, the test subset is used to confirm prediction performance of the model. The model performance is then interpreted as the mean accuracy obtained by the 10 models over the process of 10 validation rounds.

For estimating the efficacy of the model in more detail, the different statistical metrics are also utilized. These statistical measures include coefficient of determination (R^2^), root man square error (RMSE), relative root mean square (RRMSE), mean absolute error (MAE), relative squared error (RSE), and performance index (ρ). The mathematical relationship for stated chosen statistical measures is mentioned in Equations (4)–(9).

(4) $$R^{2}=1-\frac{\sum_{j=1}^{m}{\left(p_{j}-t_{j}\right)}^{2}}{\sum_{j=1}^{m}\left(t_{j}-\bar{t}\right)}$$

(5) $$\mathrm{RMSE}=\sqrt{\frac{\sum_{j=1}^{m}{\left(t_{j}-p_{j}\right)}^{2}}{n}}$$

(6) $$\mathrm{RRMSE}=\frac{1}{\left|\bar{t}\right|}\sqrt{\frac{\sum_{j=1}^{m}{\left(t_{j}-p_{j}\right)}^{2}}{n}}$$

(7) $$\mathrm{MAE}=\frac{\sum_{j=1}^{m}\left|t_{j}-p_{j}\right|}{n}$$

(8) $$\mathrm{RSE}=\frac{\sum_{j=1}^{m}{\left(p_{j}-t_{j}\right)}^{2}}{\sum_{j=1}^{m}{\left(\bar{t}-t_{j}\right)}^{2}}$$

(9) $$\rho =\frac{\mathrm{RRMSE}}{1+\sqrt{R^{2}}}$$

In the mentioned equations, the $t_{j}$ and $p_{j}$ shows the $j^{th}$ targeted and predicted outcome respectively. While the ${\bar{t}}_{j}$ and ${\bar{p}}_{i}$ are the mean values of targeted and predicted outcome for $j^{th}$ domain respectively. Additionally, m denotes the total number of instances used in modelling perspective. For a best and accurate model, higher can be the $R^{2}$ and lower the statistical errors. The $R^{2}$ enumerate the linear dependency between explanatory variables and outcome [82]. For a stronger correlation between targeted and predicted values, the $R^{2}$ can be greater than 0.8 [83]. As $R^{2}$ is insensitive towards division and multiplication of outcome to a constant, so it cannot be used exclusively as a metric of the overall efficacy of model [84]. Both, the MAE and RMSE enumerate the magnitude of the average error and have their own importance. In RMSE, the error values are squared before the calculation of average and therefore useful in interpretation of larger error. While MAE assigns low weightage to larger error values. A high RMSE represents that the large number predicted outcomes have high error and must be eliminated. Despotovic et al. [85] categorize the model as excellent and good if the RRMSE values is between 0 to 0.11 and 0.11 to 0.20 respectively. The values of *ρ* must fall between 0 to infinity. Gandomi et al. [86] recommended that for a good model the *ρ* can be less than 0.2. It is noteworthy to mention that *ρ* simultaneously covers both the factors, i.e., RRMSE and R. Hence, for a superior performance of the model, the value of *ρ* must be near to 0. Furthermore, this study also considers different statistical measures for the validation of the established model as suggested in the literature (see Table 5).

## 5. Result and Discusion

To create an AI based model, the initial and the foremost step is the selection explanatory variable which effectively and significantly explain the abrasion resistance of concrete. The input variables considered for the explanation of depth of wear in concrete are given in Equation (10).

(10) f(WD_mm)=(C, F, W, FA, CA,P,AE,A,T)

where, *C*: cement content (kg/m^3^), *F*: fly-ash (kg/m^3^), *W*: water content (kg/m^3^), *FA:* fine aggregate (kg/m^3^), *CA*: coarse aggregate (kg/m^3^), *P*: plasticizer (kg/m^3^), *AE*: air entraining agent (kg·mL/m^3^), *A*: age of concrete (days), and *T*: time of testing (min).

### 5.1. Random Forest Regression

**Figure 5:**
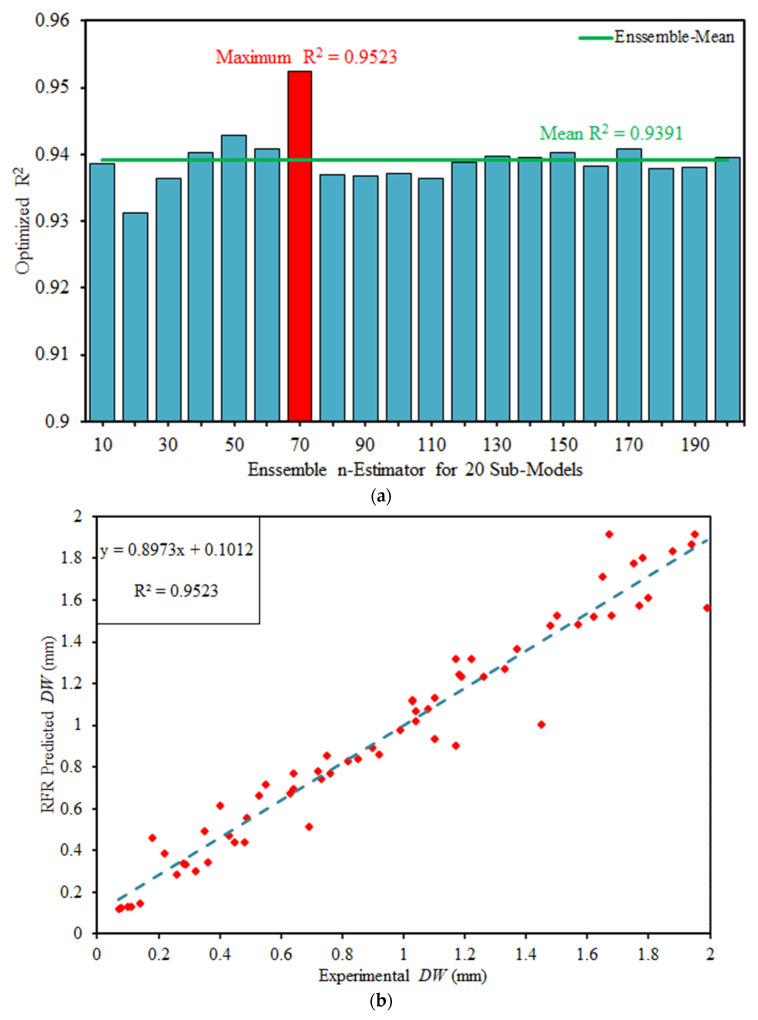

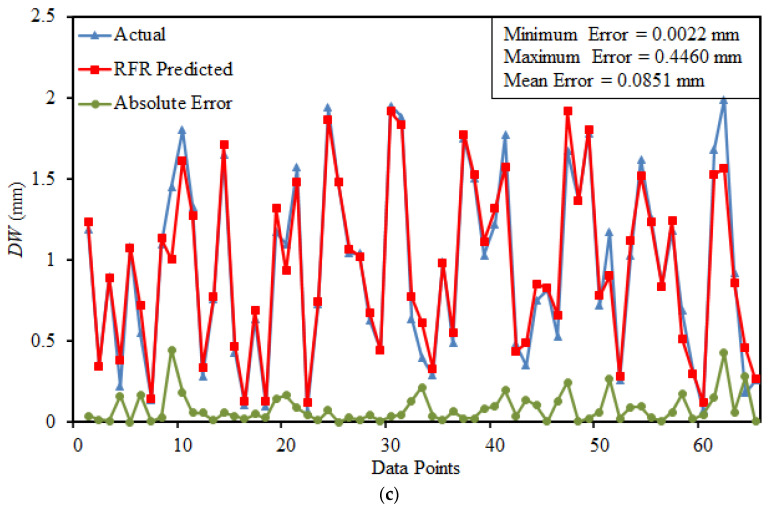
Performance of RFR model (**a**) Optimized R^2^ for 20 sub models (**b**) Regression analysis (**c**) Absolute error plot.

An ensemble bagging technique namely random forest regression (RFR) is employed for optimization of R^2^. In ensemble bagging approach every record is used at least once that improves the overall performance of the model. Twenty sub-models are generated for n-estimator ranging from 10 to 200 with an increment of 10. It can be seen in Figure 5 that the overall efficacy of the model is considerably enhanced. The RFR yields minimum, maximum and mean value of R^2^ as 0.9311, 0.9523 and 0.9391 respectively (See Figure 5a). For a generalized and reliable model, the slope of the regression line and R^2^ must be near to 1, while for an ideal scenario, this must approach to one [89]. The 7th sub-model with n-estimator equals to 70 gives an outburst performance with R^2^ equals to 0.9523 and slope of the regression line equals to 0.8973 (See Figure 5b). Thus, the results of predicted wear depth are strongly correlated with their targeted outcomes.

The absolute error plot between actual and RFR predicted outcome of 7th sub-model can be seen in Figure 5c. The model accurately captured the targeted outcome and the error values are running near the axis. The minimum, maximum and mean error values are recorded as 0.0022 mm, 0.4460 mm and 0.0851 mm. Furthermore, the occurrence of maximum error is rare.

### 5.2. Development of GEP Based Empirical Equation

**Figure 6:**
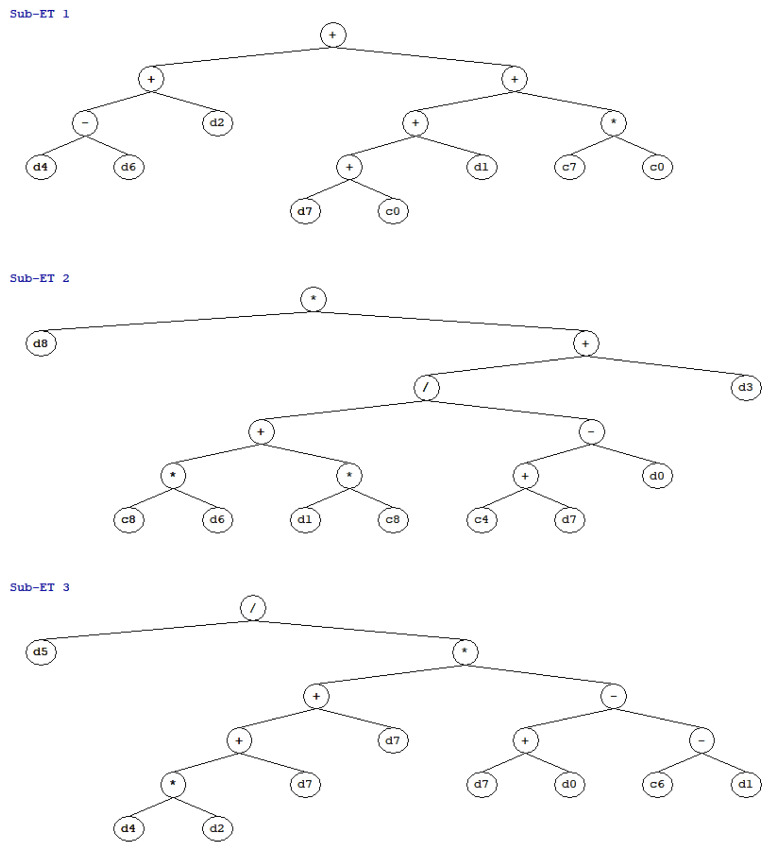
Expression trees obtained from GeneXproTool 5.0.

To get an empirical equation for finding the wear depth, GEP algorithm of the established model is presented as expression trees (ETs) in Figure 6. It can be seen that four basic arithmetical functions, i.e., +,−,× and / are used in Sub ET’s. These ETs in GEP algorithm uses different indicator like d’s and c’s for explanatory variables and constant values respectively. Where; d_0_: cement content, d_1_: fly-ash, d_2_: water content, d_3_: fine aggregate, d_4_: coarse aggregate, d_5_: plasticizer, d_6_: air entraining agent, d_7_: age of concrete, d_8_: time of testing. The ultimate expression for wear depth is shown in Equation (11) which is extracted by decoding the Sub ET-1 (*G*_1_), Sub ET-2 (*G*_2_), and Sub ET-3 (*G*_3_) that are represented as Equations (12)–(14).

(11) $$DW\;\left(mm\right)=G_{1}\times G_{2}\times G_{3}$$

where

(12) $$G_{1}=CA+AE+W+A+F+80.77$$

(13) $$G_{2}=T\times \left(\left(\frac{10.72\times \left(AE+F\right)}{-20.45+A-C}\right)+FA\right)$$

(14) $$G_{3}=\left(\frac{P}{\left(\left(CA\times W\right)+\left(2\times A\right)\right)\times \left(A+C+F-9.25\right)}\right)$$

### 5.3. Performance of GEP Model

**Figure 7:**
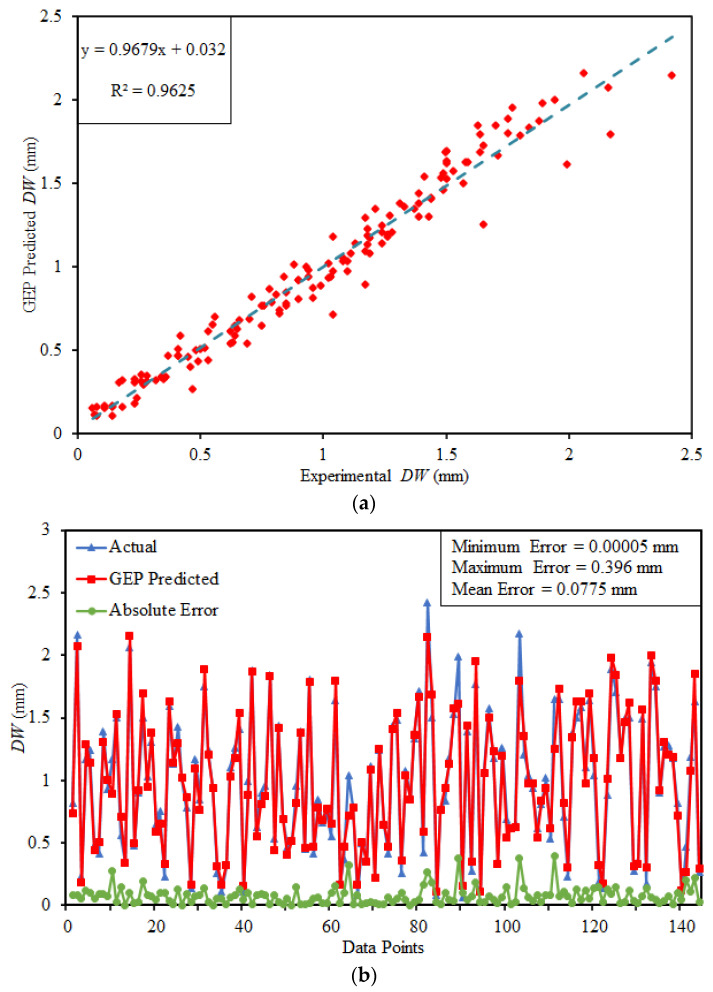
Performance of GEP model (**a**) Regression analysis (**b**) Absolute error plot.

Figure 7a illustrates the comparison between the targeted and GEP predicted values of DW for the training subset. It can be clearly seen in the Figure 7a that the established GEP model holds a strong correlation between targeted and predicted outcomes as noticeable through the slope of 0.9679 and R^2^ equals to 0.9625 for training subset. Both the regression line slope and R^2^ are close to 1 [89]. In addition, the absolute error distribution between actual and GEP predicted outcome can be seen in Figure 7b. The error runs near the axis and considerably reduced. The minimum, maximum and mean absolute error are 0.00005 mm, 0.0396 mm and 0.0775 mm. Which describes that the developed GEP model have a robust performance.

### 5.4. K-Fold Cross Validation (KFCV)

The evaluation of the models based on regression line slope and coefficient of determination is inadequate to generate and determine the optimal performance. Therefore, to analyze the robustness of the model, it was tested by K-Fold cross validation technique different statistical error checks. It is necessary for the accurate evaluation of the model, that the desired accuracy is achieved. The K-fold cross validation (KFCV) is implemented to ensure that prediction models are accurate and reliable. K-fold algorithm shuffles the data randomly to assess the accuracy of the model by minimizing the biasness in arbitrary sampling. The 10-fold CV is performed to check the reliability and generality of the model performance [75]. The entire data is split up into ten different subsets. In all ten generations of model creation and validation, it selects a different subset of data for training while testing of the model is executed with other subsets of data. In the end the average accuracy is calculated in term of statistical metrics.

**Figure 8:**
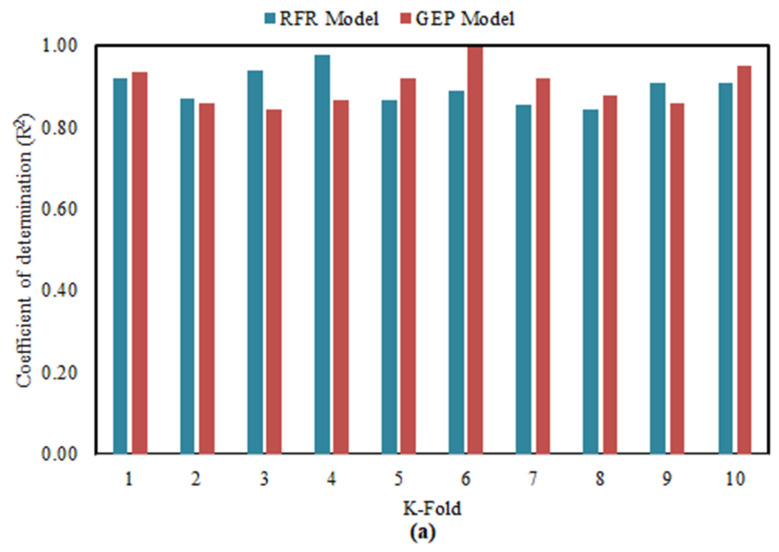

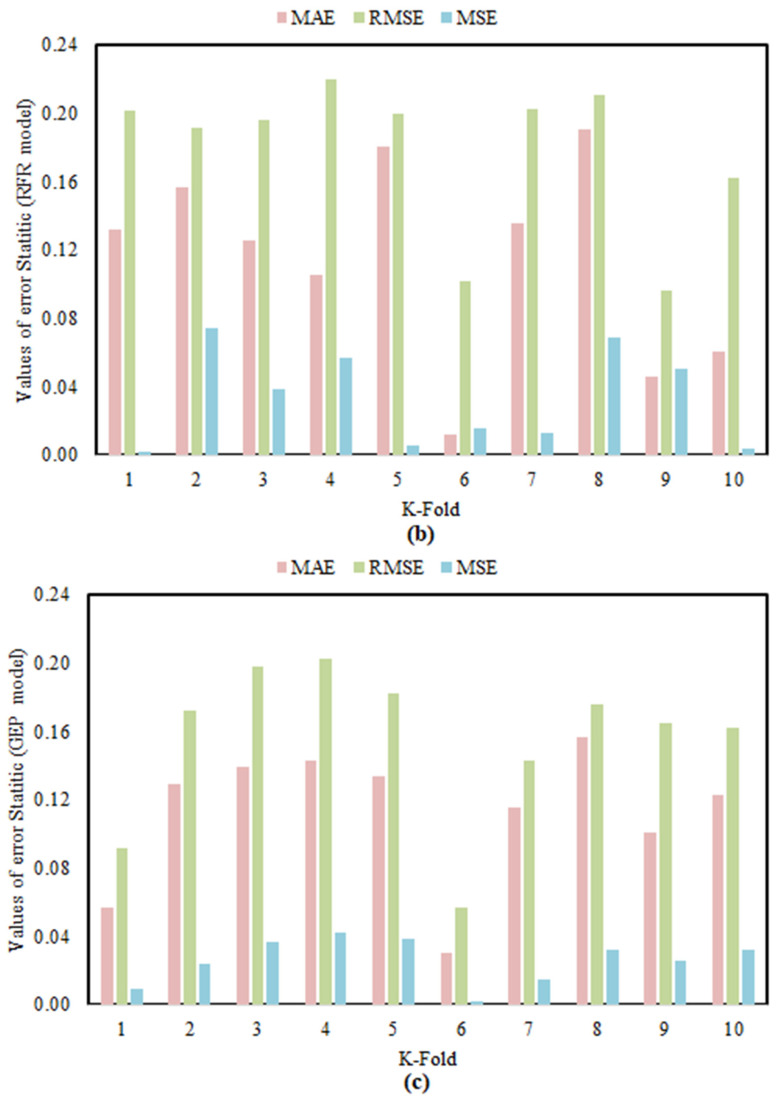
Statistical metrics used in KFCV (**a**) Coefficient of determination (R^2^), (**b**) Error statistic of RFR model (**c**) Error statistic of GEP model.

The detailed results of KFCV via the statistical metrics considered are presented in Table 6. It can be seen from Figure 8a that both the GEP and RFR models have higher mean R^2^, i.e., 0.9021 and 0.8972. It shows a strong correlation between explanatory variables and response parameter. Although the variation is noticed, i.e., the maximum and minimum value of R^2^ are 0.9953 and 0.8436 for GEP model respectively; while 0.9764 and 0.8446 for RFR model. Moreover, the results of KFCV are also evaluated through different error parameters like MSE, RMSE and MAE. As shown in Figure 8b,c the mean value of MAE, RMSE and MSE for GEP model is 0.11282 mm, 0.15474 mm and 0.02584 mm respectively; and 0.1145 mm, 0.17816 mm, and 0.03292 mm respectively for RFR model. The lesser values of error statistics dictate the accurateness and generalization of the models.

The statistical error checks are also exercised to assess the performance of the models. The values of these checks, for the estimation of wear depth are summarized in Table 7. For both the model, RMSE, MAE, and RSE are considerably lower which signifies the accurateness and generalization capacity of models. The MAE for RF and GEP model are 0.08511 mm and 0.07361 respectively. Which are lower than RMSE values satisfying the analysis criteria stated in Section 4.3. Based on the RRMSE, GEP model shows an excellent performance as it is lesser than 0.11, i.e., 0.09473 while for RF model RRMSE is 0.13420 that falls between 0.11 and 0.22. Hence, the RF model can be categorized as good. The results of performance index *ρ* for both the model lesser than 0.2 and close to 0, representing that the projected models are reliable and have the capacity to accurately predict the outcome.

The values of the statistical metrics for the external validation of the models recommended by different authors are listed in Table 8. For both models, the k and k’ are closer to 1 satisfying the criteria recommended by Golbraikh and Tropsha [87]. The R_m_ for both models is also greater than 0.5 and fulfil the recommended criteria by Roy and Roy [88]. Thus, both models are valid having a robust performance and are not a simple correlation between explanatory variables and response parameter.

**Figure 9:**
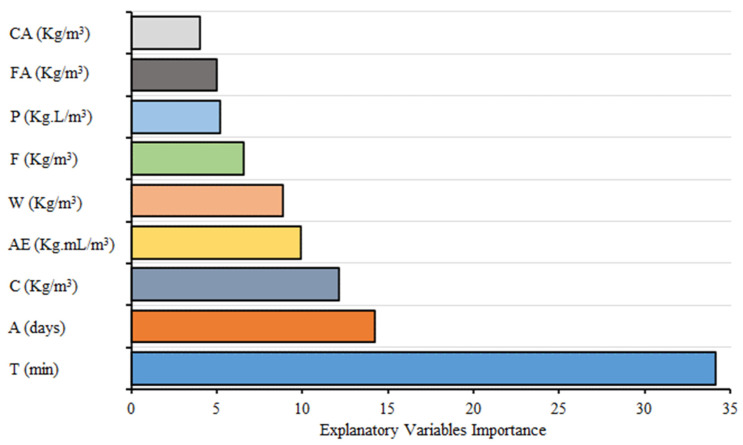
Importance of explanatory variables on the wear depth of concrete.

Furthermore, the influence of considered explanatory variables on depth of wear is analyzed through permutation feature analysis using python. As shown in Figure 9 the considered explanatory variables have an immense impact on the estimation of wear depth of concrete following the order: T (34.16%) > A (14.22%) > C (12.13%) > AE (9.914%) > W (8.834%) > F (6.551%) > P (5.221%) > FA (4.985%) > CA (3.986%). Thus, every explanatory variable is important in estimating the wear depth of concrete. Moreover, cement, age of specimen and time of testing are the most influential parameters that affect the abrasion as well as compressive strength of concrete.

## 6. Limitation and Recommendation for Future Study

In spite of the fact that the research included a detailed analysis supported by an enormous data of experimental records collected from literature and an extensive algorithm is applied with evaluation, the limitations and constraints of the research should be discussed. The performance and reliability of the model depends on the total number of instances used for modelling. As stated by Frank and Todeschini [89], the ratio between the number of records to the number of explanatory variables must equal to 3 and preferably more than 5 [89]. This research works satisfies recommended criteria. Though, the number of experimental records equal to 216 is an uncertain limitation of the research. It is worth mentioning that this research is limited to estimate abrasion resistance of concrete and consider nine different explanatory variables. It should be extended to predict other mechanical properties of concrete like tensile strength, toughness, corrosion and durability by using a huge data base that consider extensive number of explanatory variables. Additionally, the performance of concrete to elevated temperature should be estimated.

Furthermore, other machine learning techniques, such as support vector machine (SVM), particle swarm optimization (PSO), back tracking search algorithm (BSA), cuckoo search algorithm (CSA), AdaBoost and gradient boosting and evolutionary algorithm like multi-expression programming (MEP) be used. However, this study does not only concentrate on algorithmic-based methods, but also on programming based on GEP, indicating the diversity of this research. The multi-stage genetic programming technique can also be used to obtain extreme accuracy in both actual and predicted results. It is also suggested to develop models for concrete modified with various pozzolanic materials like bagasse ash and rice husk ash. Moreover, the models can be executed for the concrete incorporating the effect of various fibers such as glass fibers, jute fibers, nylon fibers, steel fibers and polypropylene fibers.

## 7. Conclusions

In this research, a machine learning technique namely gene expression programming (GEP) and an ensemble algorithm known as random forest regression (RFR) along with K-fold cross validation (KFCV) algorithm are operated to determine the depth of wear (*DW*) of fly-ash based concrete by utilizing 216 experimental records.

- The results disclose that the RFR and GEP model can precisely and accurately estimate the *DW* exclusive of any prior assumption. Moreover, the *DW* estimation from GEP model is better than RFR based model. GEP technique delivers a simplified formula of *DW* with considerably greater accuracy between experimental and predicted outcome. This shows the diverse nature of GEP technique as it has space for non-linear and linear data.
- The performance of both models was testified via statistical metrics like R^2^, MAE, RMSE, RSE, RRMSE and performance index (*ρ*). The analysis of all metrics reveals that both the models deliver an outburst performance. The R^2^ of RFR and GEP model comes out to be 0.9523 and 0.9667 respectively. Since the *ρ* of predicted *DW* by RFR and GEP are lesser than 0.2 that is 0.0679 and 0.0501, respectively; so, both models can be categorized as good models. The models also meet the external validation criterion suggested in the previous literature.
- The validation via KFCV reveals that the model’s variables are highly correlated and accurate having a minimal error statistic between predicted *DW* and experimental results.
- The sensitivity analysis via GEP based formulation shows that the considered explanatory variables have an immense impact on the estimation of wear depth of concrete following the order: T (34.16%) > A (14.22%) > C (12.13%) > AE (9.914%) > W (8.834%) > F (6.551%) > P (5.221%) > FA (4.985%) > CA (3.986%).
- The simplified mathematical expression delivered by GEP algorithm for predicting the *DW* of fly-ash based concrete are much simpler. The established GEP equation is recommended to be utilized in the routine-based design practices rather than performing time-consuming and laborious experimental tests. It is noteworthy to mark that the projected equation is generally capable to predict the *DW* within the vast range of explanatory variables exercised during formulation. In addition, the results can be used to check the applicability of different mix design ratio of fly-ash concrete. The site engineer can design the required mix ratio keeping the cost of concrete as low as required with little or no help from the consultants.

According to the conclusions of the current study, it is valuable to mention that the artificial techniques (AI) are greatly robust and accessible tools to resolve the problems involving the complicated mechanism mostly in materials engineering. The simple mathematical expression can be generalized to new fresh data. Thus, it can lead to construction of sustainable structures that uses a hazardous material like fly-ash, which results in lower consumption of energy and lesser cost of construction.

## Figures and Tables

**Table 1:** Effect of waste material on properties of concrete.

S.No	Waste Material	Property Studied	Conclusive Remarks	Reference
1	Super fine slag (SFS), nano-SiO_2_ (NS), fly ash (FA)	Abrasion resistance and microstructure of concrete.	Maximum enhancement in strength with 82%, 73% and 68% for surface mortar layer and 20%, 16% and 13% for concrete.	[22]
2	Shredded plastic waste bags	Density, abrasion resistance, compressive strength, workability and flexural strength.	Abrasion resistance, impact resistance and energy absorption increase. However compressive, flexural strength decreases.	[23]
3	Crushed granite coarse aggregate	Compressive strength and abrasion resistance	Coarse aggregate with 45% by mass content of cement show better performance as compared to fine aggregate with 28.7%.	[24]
4	Silica fume and PVA fiber	Tensile and compressive strength, abrasion resistance, volume stability and drying shrinkage	Addition of silica fume and PVA fiber shows enhancement in compressive strength and abrasion resistance.	[25]
5	Granite waste (GW)	Compressive strength and abrasion resistance	Significant enhancement is observed in compressive and abrasion resistance of self-compacting concrete.	[26]
6	Waste glass fiber (WGF)	Mechanical and abrasion resistance	Addition of 2% WFG yield maximum strength mechanism of rolled compacted concrete.	[27]
7	Nano size particles (silicon dioxide and Chromium oxide)	Abrasion resistance and compressive strength	Improvement in abrasion resistance is observed in both cured saturated lime water and in water. However, sample containing SiO_2_ show much more abrasion resistance as compared to other specimens.	[28]
8	Polypropylene fibers (PP), nano-silica (SiO_2_), and nano titanium oxide (TiO_2_)	Abrasion resistance of pavement concrete	Nanoparticles show maximum improvement. Furthermore, titanium oxide (TiO_2_) show an overall enhancement response in specimen of pavement concrete.	[29]
9	Waste polypropylene fibers (PP) and palm oil fuel ash (POFA)	Abrasion and skid resistance of pavement concrete	Intrusion of PP show decrease in compressive strength by 17% with enhancement in abrasion resistance by 25% is observed.	[16]
10	Copper slag as fine aggregate	Copper slag concrete	Improvement is observed by using cooper slag in concrete.	[33]

**Table 2:** Machine learning algorithm used by researchers.

S.No	Concrete Type	Properties	Techniques	References
1	Normal concrete	Compressive strength	Genetic programming	[37]
			ANN	[38]
2	High-performance concrete	Compressive strength	Random forest	[39]
			ANN	[40,41,42]
			M5P	[43]
			Gene expression programming	[44]
3	Silica fume concrete	Compressive strength	Hybrid ANN	[45]
			Biogeography-based programming (BBP)	[46]
			ANN and ANFIS	[47]
4	Self-compacting concrete	Modulus of Elasticity	Biogeography-based programming (BBP)	[48]
		Compressive strength	Artificial neuron network (ANN) and gene expression programming (GEP)	[49]
5	Recycled aggregate concrete	Modulus of Elasticity	M5P	[50]
6	Concrete filled steel tube	Compressive strength	GEP	[51]
7	High-performance concrete	Compressive strength	BANN	[52]
			GBANN	
			Adaptive boosting	[53]
			RF	[39]
			Gradient tree boosting	[54]
8	Recycled aggregate concrete	Modulus of Elasticity	RF+SVM	[55]
9	Corrosion of concrete sewer	Microbially induced concrete corrosion	Bagging/Boosting MLPNN/RBFNN/CHAID/CART	[56]
10	corrosion of concrete sewer	Microbially induced concrete corrosion	Ensemble RF	[56]
11	RC panels	Failure modes	GBML	[57]
12	Lightweight self-compacting concrete	Compressive strength	RF	[58]
13	Concrete filled steel tube	Bearing capacity	Gene expression programming	[59]
14	Concrete Containing Waste Material	Surface Chloride Concentration	Gene expression programming, Artificial neural network, Decision tree	[60]
15	Concrete with high calcium fly ash	Depth of wear of cement composite	Artificial neuron network	[20]
16	Concrete	Abrasive wear	Artificial neuron network and general linear model	[61]
17	Beam reinforced with FRP bars	Flexural strength	Gene expression programming	[62]
18	Fiber concrete beam	Shear strength	Particle Swarm Optimization	[63]
19	Fresh concrete	Yield stress and plastic viscosity	Particle swarm optimization and least squares support vector machine and	[64]
20	Ultra-high performance propylene-fiberious cementicious composites (UHPPFCC)	Compressive strength and impact energy	Taguchi approach	[65]

**Table 3:** Coefficient of correlation for explanatory variables.

	C	F	W	FA	CA	P	AE	A	T	DW
C	1									
F	−0.787	1								
W	−0.525	0.461	1							
FA	0.754	−0.645	−0.732	1						
CA	0.774	−0.666	−0.689	0.750	1					
P	−0.379	0.282	0.750	−0.727	−0.729	1				
AE	−0.524	0.532	−0.136	−0.174	−0.362	−0.161	1			
A	0	0	0	0	0	0	0	1		
T	0	0	0	0	0	0	0	0	1	
DW	−0.032	0.032	0.046	−0.022	−0.039	−0.052	−0.005	−0.191	0.737	1

**Table 4:** Statistic of response and explanatory parameters.

Parameters	Kurtosis	Skewness	Mean	Median	Mode	Minimum	Maximum	SD
Explanatory								
C (kg/m^3^)	−0.97	−0.54	346.17	351.50	398.00	259.00	398.00	49.64
F (kg/m^3^)	−1.03	0.59	47.00	35.50	0.00	0.00	139.00	52.23
W (kg/m^3^)	−1.35	0.03	130.67	131.00	123.00	123.00	139.00	5.60
FA (kg/m^3^)	−1.43	0.15	695.67	694.00	715.00	677.00	715.00	14.00
CA (kg/m^3^)	−1.55	0.49	1210.67	1194.50	1259.00	1172.00	1264.00	37.62
P (kg·L/m^3^)	−1.51	0.00	2.80	2.80	2.70	2.70	2.90	0.08
AE (kg·mL/m^3^)	−0.55	0.78	325.00	315.00	280.00	270.00	420.00	50.70
A (days)	−1.51	0.61	161.33	91.00	28.00	28.00	365.00	146.63
T (min)	−1.22	0.00	32.50	32.50	5.00	5.00	60.00	17.30
Response								
DW (mm)	−0.89	0.11	1.00	1.03	1.50	0.05	2.42	0.56

**Table 5:** Statistical metrics suggested in literature.

Equations	Condition	Recommended by
$k=\frac{\sum_{j=1}^{m}\left(t_{j}\times p_{j}\right)}{t_{j}^{2}}$	0.85 < *k* < 1.15	[87]
$k{}^{\prime }=\frac{\sum_{j=1}^{m}\left(t_{j}\times p_{j}\right)}{p_{j}^{2}}$	0.85 < *k*′ < 1.15	[87]
$R_{m}=R^{2}\times \left(1-\sqrt{\left|R^{2}-R_{0}^{2}\right|}\right)$	*R_m_* > 0.5	[88]
where		
$\;\;R_{o}^{2}=1-\frac{\sum_{j=1}^{m}{\left(p_{j}-t_{j}^{o}\right)}^{2}}{\sum_{j=1}^{m}{\left(p_{j}-\bar{p_{j}^{o}}\right)}^{2}\;};\;t_{j}^{o}=k\times p_{j}$	$\;\;R_{o}^{2}\;≅1$	
$R_{o}^{\prime }{}^{2}=1-\frac{\sum_{j=1}^{m}{\left(t_{j}-p_{j}^{o}\right)}^{2}}{\sum_{j=1}^{m}{\left(t_{j}-\bar{t_{j}^{o}}\right)}^{2}\;};\;p_{j}^{o}=k{}^{\prime }\times t_{j}$	$\;R_{o}^{\prime }{}^{2}\;≅1$	

**Table 6:** Summary of statistical metrics considered in K-Fold cross validation.

K-Fold	RFR Model				GEP Model			
	R^2^	MAE (mm)	RMSE (mm)	MSE (mm)	R^2^	MAE (mm)	RMSE (mm)	MSE (mm)
1	0.9186	0.13195	0.20130	0.00169	0.9354	0.05727	0.09130	0.00916
2	0.8693	0.15670	0.19173	0.07457	0.8592	0.12950	0.17166	0.02429
3	0.9393	0.12563	0.19590	0.03876	0.8436	0.13900	0.19770	0.03682
4	0.9764	0.10530	0.21984	0.05693	0.8656	0.14264	0.20269	0.04257
5	0.8654	0.18059	0.19959	0.00561	0.9198	0.13376	0.18250	0.03881
6	0.8892	0.01179	0.10130	0.01567	0.9953	0.03065	0.05653	0.00172
7	0.8544	0.13570	0.20250	0.01342	0.9174	0.11553	0.14255	0.01520
8	0.8446	0.19063	0.21056	0.06876	0.8762	0.15620	0.17543	0.03204
9	0.9065	0.04580	0.09640	0.05016	0.8575	0.10068	0.16461	0.02551
10	0.9085	0.06039	0.16246	0.00364	0.9509	0.12297	0.16246	0.03225
Maximum	0.9764	0.19063	0.21984	0.07457	0.9953	0.15620	0.20269	0.04257
Minimum	0.8446	0.01179	0.09640	0.00169	0.8436	0.03065	0.05653	0.00172
Mean	0.8972	0.11445	0.17816	0.03292	0.9021	0.11282	0.15474	0.02584

**Table 7:** Summary of statistical error checks and performance index.

Developed Models	R^2^	MAE (mm)	RMSE (mm)	RRMSE	RSE (mm)	Sigma
GEP	0.9667	0.07361	0.10631	0.09947	0.033263	0.050157
RFR	0.9523	0.08511	0.13420	0.13420	0.05062	0.067919

**Table 8:** Summary of Statistical metrics suggested in literature.

Suggested Metric	RFR Model	GEP Model
k	0.97635	0.99213
$k{}^{\prime }$	1.0107	1.0000
$R_{m}$	0.74995	0.791307
$\;\;R_{o}^{2}$	0.9975	0.999715
$R_{o}^{\prime }{}^{2}$	0.9491	0.96597

## Data Availability

The data presented in this article are available within the article.

## Appendix A

**Table A1:** Database of experimental results.

Sr.No.	Cement (kg/m^3^)	Fly Ash (kg/m^3^)	Water (kg/m^3^)	Fine Aggregate (kg/m^3^)	Coarse Aggregate (kg/m^3^)	Plasticizer (kg/m^3^)	Air Entraining (g/m^3^)	Age (Days)	Time of Testing (mins)	Depth of Wear (mm)
1	398	0	123	715	1259	2.7	280	28	5	0.11
2	398	0	123	715	1259	2.7	280	28	10	0.26
3	398	0	123	715	1259	2.7	280	28	15	0.64
4	398	0	123	715	1259	2.7	280	28	20	1.04
5	398	0	123	715	1259	2.7	280	28	25	1.17
6	398	0	123	715	1259	2.7	280	28	30	1.45
7	398	0	123	715	1259	2.7	280	28	35	1.65
8	398	0	123	715	1259	2.7	280	28	40	1.88
9	398	0	123	715	1259	2.7	280	28	45	1.99
10	398	0	123	715	1259	2.7	280	28	50	2.17
11	398	0	123	715	1259	2.7	280	28	55	2.28
12	398	0	123	715	1259	2.7	280	28	60	2.42
13	397	0	125	712	1264	2.7	330	28	5	0.1
14	397	0	125	712	1264	2.7	330	28	10	0.26
15	397	0	125	712	1264	2.7	330	28	15	0.41
16	397	0	125	712	1264	2.7	330	28	20	0.63
17	397	0	125	712	1264	2.7	330	28	25	0.75
18	397	0	125	712	1264	2.7	330	28	30	0.88
19	397	0	125	712	1264	2.7	330	28	35	1.04
20	397	0	125	712	1264	2.7	330	28	40	1.21
21	397	0	125	712	1264	2.7	330	28	45	1.33
22	397	0	125	712	1264	2.7	330	28	50	1.5
23	397	0	125	712	1264	2.7	330	28	55	1.67
24	397	0	125	712	1264	2.7	330	28	60	1.85
25	375	0	135	682	1182	2.9	270	28	5	0.23
26	375	0	135	682	1182	2.9	270	28	10	0.46
27	375	0	135	682	1182	2.9	270	28	15	0.69
28	375	0	135	682	1182	2.9	270	28	20	0.82
29	375	0	135	682	1182	2.9	270	28	25	1.01
30	375	0	135	682	1182	2.9	270	28	30	1.11
31	375	0	135	682	1182	2.9	270	28	35	1.28
32	375	0	135	682	1182	2.9	270	28	40	1.39
33	375	0	135	682	1182	2.9	270	28	45	1.57
34	375	0	135	682	1182	2.9	270	28	50	1.75
35	375	0	135	682	1182	2.9	270	28	55	1.89
36	375	0	135	682	1182	2.9	270	28	60	2.06
37	328	72	139	695	1207	2.9	300	28	5	0.14
38	328	72	139	695	1207	2.9	300	28	10	0.36
39	328	72	139	695	1207	2.9	300	28	15	0.52
40	328	72	139	695	1207	2.9	300	28	20	0.7
41	328	72	139	695	1207	2.9	300	28	25	0.92
42	328	72	139	695	1207	2.9	300	28	30	1.08
43	328	72	139	695	1207	2.9	300	28	35	1.24
44	328	72	139	695	1207	2.9	300	28	40	1.39
45	328	72	139	695	1207	2.9	300	28	45	1.62
46	328	72	139	695	1207	2.9	300	28	50	1.78
47	328	72	139	695	1207	2.9	300	28	55	1.96
48	328	72	139	695	1207	2.9	300	28	60	2.16
49	259	139	133	677	1172	2.8	350	28	5	0.14
50	259	139	133	677	1172	2.8	350	28	10	0.34
51	259	139	133	677	1172	2.8	350	28	15	0.5
52	259	139	133	677	1172	2.8	350	28	20	0.66
53	259	139	133	677	1172	2.8	350	28	25	0.85
54	259	139	133	677	1172	2.8	350	28	30	1.02
55	259	139	133	677	1172	2.8	350	28	35	1.18
56	259	139	133	677	1172	2.8	350	28	40	1.33
57	259	139	133	677	1172	2.8	350	28	45	1.5
58	259	139	133	677	1172	2.8	350	28	50	1.74
59	259	139	133	677	1172	2.8	350	28	55	1.88
60	259	139	133	677	1172	2.8	350	28	60	2.05
61	320	71	129	693	1180	2.8	420	28	5	0.18
62	320	71	129	693	1180	2.8	420	28	10	0.32
63	320	71	129	693	1180	2.8	420	28	15	0.54
64	320	71	129	693	1180	2.8	420	28	20	0.64
65	320	71	129	693	1180	2.8	420	28	25	0.9
66	320	71	129	693	1180	2.8	420	28	30	1.03
67	320	71	129	693	1180	2.8	420	28	35	1.18
68	320	71	129	693	1180	2.8	420	28	40	1.33
69	320	71	129	693	1180	2.8	420	28	45	1.49
70	320	71	129	693	1180	2.8	420	28	50	1.65
71	320	71	129	693	1180	2.8	420	28	55	1.8
72	320	71	129	693	1180	2.8	420	28	60	1.95
73	398	0	123	715	1259	2.7	280	91	5	0.08
74	398	0	123	715	1259	2.7	280	91	10	0.23
75	398	0	123	715	1259	2.7	280	91	15	0.43
76	398	0	123	715	1259	2.7	280	91	20	0.55
77	398	0	123	715	1259	2.7	280	91	25	0.72
78	398	0	123	715	1259	2.7	280	91	30	0.94
79	398	0	123	715	1259	2.7	280	91	35	1.13
80	398	0	123	715	1259	2.7	280	91	40	1.27
81	398	0	123	715	1259	2.7	280	91	45	1.37
82	398	0	123	715	1259	2.7	280	91	50	1.5
83	398	0	123	715	1259	2.7	280	91	55	1.64
84	398	0	123	715	1259	2.7	280	91	60	1.8
85	397	0	125	712	1264	2.7	330	91	5	0.08
86	397	0	125	712	1264	2.7	330	91	10	0.23
87	397	0	125	712	1264	2.7	330	91	15	0.45
88	397	0	125	712	1264	2.7	330	91	20	0.62
89	397	0	125	712	1264	2.7	330	91	25	0.75
90	397	0	125	712	1264	2.7	330	91	30	0.9
91	397	0	125	712	1264	2.7	330	91	35	1.03
92	397	0	125	712	1264	2.7	330	91	40	1.12
93	397	0	125	712	1264	2.7	330	91	45	1.27
94	397	0	125	712	1264	2.7	330	91	50	1.41
95	397	0	125	712	1264	2.7	330	91	55	1.5
96	397	0	125	712	1264	2.7	330	91	60	1.63
97	375	0	135	682	1182	2.9	270	91	5	0.14
98	375	0	135	682	1182	2.9	270	91	10	0.29
99	375	0	135	682	1182	2.9	270	91	15	0.49
100	375	0	135	682	1182	2.9	270	91	20	0.75
101	375	0	135	682	1182	2.9	270	91	25	0.96
102	375	0	135	682	1182	2.9	270	91	30	1.1
103	375	0	135	682	1182	2.9	270	91	35	1.24
104	375	0	135	682	1182	2.9	270	91	40	1.39
105	375	0	135	682	1182	2.9	270	91	45	1.46
106	375	0	135	682	1182	2.9	270	91	50	1.58
107	375	0	135	682	1182	2.9	270	91	55	1.68
108	375	0	135	682	1182	2.9	270	91	60	1.77
109	328	72	139	695	1207	2.9	300	91	5	0.06
110	328	72	139	695	1207	2.9	300	91	10	0.26
111	328	72	139	695	1207	2.9	300	91	15	0.41
112	328	72	139	695	1207	2.9	300	91	20	0.62
113	328	72	139	695	1207	2.9	300	91	25	0.79
114	328	72	139	695	1207	2.9	300	91	30	0.94
115	328	72	139	695	1207	2.9	300	91	35	1.11
116	328	72	139	695	1207	2.9	300	91	40	1.27
117	328	72	139	695	1207	2.9	300	91	45	1.44
118	328	72	139	695	1207	2.9	300	91	50	1.53
119	328	72	139	695	1207	2.9	300	91	55	1.65
120	328	72	139	695	1207	2.9	300	91	60	1.75
121	259	139	133	677	1172	2.8	350	91	5	0.05
122	259	139	133	677	1172	2.8	350	91	10	0.17
123	259	139	133	677	1172	2.8	350	91	15	0.35
124	259	139	133	677	1172	2.8	350	91	20	0.53
125	259	139	133	677	1172	2.8	350	91	25	0.76
126	259	139	133	677	1172	2.8	350	91	30	0.9
127	259	139	133	677	1172	2.8	350	91	35	1.04
128	259	139	133	677	1172	2.8	350	91	40	1.18
129	259	139	133	677	1172	2.8	350	91	45	1.31
130	259	139	133	677	1172	2.8	350	91	50	1.48
131	259	139	133	677	1172	2.8	350	91	55	1.64
132	259	139	133	677	1172	2.8	350	91	60	1.7
133	320	71	129	693	1180	2.8	420	91	5	0.1
134	320	71	129	693	1180	2.8	420	91	10	0.27
135	320	71	129	693	1180	2.8	420	91	15	0.53
136	320	71	129	693	1180	2.8	420	91	20	0.64
137	320	71	129	693	1180	2.8	420	91	25	0.82
138	320	71	129	693	1180	2.8	420	91	30	0.99
139	320	71	129	693	1180	2.8	420	91	35	1.1
140	320	71	129	693	1180	2.8	420	91	40	1.26
141	320	71	129	693	1180	2.8	420	91	45	1.39
142	320	71	129	693	1180	2.8	420	91	50	1.5
143	320	71	129	693	1180	2.8	420	91	55	1.59
144	320	71	129	693	1180	2.8	420	91	60	1.71
145	398	0	123	715	1259	2.7	280	365	5	0.07
146	398	0	123	715	1259	2.7	280	365	10	0.19
147	398	0	123	715	1259	2.7	280	365	15	0.28
148	398	0	123	715	1259	2.7	280	365	20	0.37
149	398	0	123	715	1259	2.7	280	365	25	0.42
150	398	0	123	715	1259	2.7	280	365	30	0.56
151	398	0	123	715	1259	2.7	280	365	35	0.71
152	398	0	123	715	1259	2.7	280	365	40	0.84
153	398	0	123	715	1259	2.7	280	365	45	1.08
154	398	0	123	715	1259	2.7	280	365	50	1.19
155	398	0	123	715	1259	2.7	280	365	55	1.17
156	398	0	123	715	1259	2.7	280	365	60	1.44
157	397	0	125	712	1264	2.7	330	365	5	0.08
158	397	0	125	712	1264	2.7	330	365	10	0.24
159	397	0	125	712	1264	2.7	330	365	15	0.35
160	397	0	125	712	1264	2.7	330	365	20	0.49
161	397	0	125	712	1264	2.7	330	365	25	0.63
162	397	0	125	712	1264	2.7	330	365	30	0.76
163	397	0	125	712	1264	2.7	330	365	35	0.85
164	397	0	125	712	1264	2.7	330	365	40	0.96
165	397	0	125	712	1264	2.7	330	365	45	1.04
166	397	0	125	712	1264	2.7	330	365	50	1.17
167	397	0	125	712	1264	2.7	330	365	55	1.28
168	397	0	125	712	1264	2.7	330	365	60	1.36
169	375	0	135	682	1182	2.9	270	365	5	0.14
170	375	0	135	682	1182	2.9	270	365	10	0.22
171	375	0	135	682	1182	2.9	270	365	15	0.18
172	375	0	135	682	1182	2.9	270	365	20	0.4
173	375	0	135	682	1182	2.9	270	365	25	0.57
174	375	0	135	682	1182	2.9	270	365	30	0.64
175	375	0	135	682	1182	2.9	270	365	35	0.73
176	375	0	135	682	1182	2.9	270	365	40	0.78
177	375	0	135	682	1182	2.9	270	365	45	1.04
178	375	0	135	682	1182	2.9	270	365	50	1.11
179	375	0	135	682	1182	2.9	270	365	55	1.26
180	375	0	135	682	1182	2.9	270	365	60	1.43
181	328	72	139	695	1207	2.9	300	365	5	0.11
182	328	72	139	695	1207	2.9	300	365	10	0.35
183	328	72	139	695	1207	2.9	300	365	15	0.48
184	328	72	139	695	1207	2.9	300	365	20	0.6
185	328	72	139	695	1207	2.9	300	365	25	0.81
186	328	72	139	695	1207	2.9	300	365	30	0.93
187	328	72	139	695	1207	2.9	300	365	35	1.11
188	328	72	139	695	1207	2.9	300	365	40	1.3
189	328	72	139	695	1207	2.9	300	365	45	1.57
190	328	72	139	695	1207	2.9	300	365	50	1.71
191	328	72	139	695	1207	2.9	300	365	55	1.84
192	328	72	139	695	1207	2.9	300	365	60	1.94
193	259	139	133	677	1172	2.8	350	365	5	0.18
194	259	139	133	677	1172	2.8	350	365	10	0.47
195	259	139	133	677	1172	2.8	350	365	15	0.46
196	259	139	133	677	1172	2.8	350	365	20	0.62
197	259	139	133	677	1172	2.8	350	365	25	0.73
198	259	139	133	677	1172	2.8	350	365	30	0.9
199	259	139	133	677	1172	2.8	350	365	35	1.03
200	259	139	133	677	1172	2.8	350	365	40	1.19
201	259	139	133	677	1172	2.8	350	365	45	1.22
202	259	139	133	677	1172	2.8	350	365	50	1.37
203	259	139	133	677	1172	2.8	350	365	55	1.49
204	259	139	133	677	1172	2.8	350	365	60	1.5
205	320	71	129	693	1180	2.8	420	365	5	0.11
206	320	71	129	693	1180	2.8	420	365	10	0.28
207	320	71	129	693	1180	2.8	420	365	15	0.41
208	320	71	129	693	1180	2.8	420	365	20	0.65
209	320	71	129	693	1180	2.8	420	365	25	0.85
210	320	71	129	693	1180	2.8	420	365	30	1.02
211	320	71	129	693	1180	2.8	420	365	35	1.18
212	320	71	129	693	1180	2.8	420	365	40	1.24
213	320	71	129	693	1180	2.8	420	365	45	1.35
214	320	71	129	693	1180	2.8	420	365	50	1.49
215	320	71	129	693	1180	2.8	420	365	55	1.67
216	320	71	129	693	1180	2.8	420	365	60	1.81
